# Management of acquired bronchobiliary fistula: 3 case reports and a literature review

**DOI:** 10.1186/1749-8090-2-52

**Published:** 2007-12-03

**Authors:** Hatice Eryigit, Selahattin Oztas, Senol Urek, Guven Olgac, Melahat Kurutepe, Cemal Asim Kutlu

**Affiliations:** 1 Department of Thoracic Surgery, Sureyyapasa Chest Diseases and Thoracic Surgery Teaching and Research Hospital, Istanbul, Turkey; 2 Department of Chest Diseases, Sureyyapasa Chest Diseases and Thoracic Surgery Teaching and Research Hospital, Istanbul, Turkey

## Abstract

Bronchobiliary fistula (BBF), which often presents with bilioptysis, is an abnormal communication between the bronchial system and biliary tree. It is a complication associated with a high mortality rate and requires a well-planned management strategy. Although hydatid disease is still the leading cause, extensive surgical interventions and invasive procedures of the liver have altered the profile of patients in recent decades. This paper presents 3 cases of BBF and reviews the literature regarding the treatment options generally mandated by clinical presentation and the underlying disease.

## Introduction

Bronchobiliary fistula (BBF), which requires aggressive treatment to decrease the mortality and morbidity rates, is an abnormal communication between the biliary channels and the bronchial tree. It occurs as a congenital malformation, but most patients have a history of liver pathology that involves the lungs [1]. In the literature, the number of reported cases of BBF has increased significantly in recent years; however, management of this complication has not been extensively discussed. In this paper, we report 3 cases and review the literature regarding the management of BBF.

### Case 1

A 35-year-old man was admitted to our clinic with cough, dyspnea, yellow-green sputum, and mild jaundice. His past medical history revealed that he had undergone laparotomy twice 36 and 2 months ago, due to hydatid disease of the liver. Blood analysis, otherwise normal, revealed increased bilirubin levels (total bilirubin, 3.2 mg/dl and conjuged bilirubin, 0.41 mg/dl), and sputum analysis revealed a total bilirubin level of 2.08 mg/dl and conjuged bilirubin level of 0.11 mg/dl. A chest x-ray and computerized tomography scan showed patchy densities over the right lung. MR cholangiography revealed no pathology, except for mild dilatation of the intrahepatic biliary channels.

Right thoracotomy was performed through the 7th intercostal space. Following pneumolysis, phrenotomy was performed; the adhesions between the diaphragm and liver were divided to expose the fistula tract, which was subsequently excised. Infected tissue in the dome was debrided, and bile leakage was carefully examined. A drain was inserted below the diaphragm, which was closed by imbrication. Apart from localized visceral pleural damage, the lower lobe appeared healthy and capable of full expansion. Therefore, resection was not performed; instead, the damaged area was primarily repaired and buttressed with a pleural flap. The chest drain was removed on the 1st postoperative day, and the subdiaphragmatic drain was removed on the 11th day. Clinical follow-up has been continuing since 12 months without any complaint.

### Case 2

A 20-year-old man was transferred to our unit from the general surgery department where he had undergone urgent laparotomy for a gunshot wound 13 days ago. On exploration, it was detected that the bullet had crossed the dome of the liver and the diaphragm and had entered the thorax. The liver and diaphragm were repaired, and a chest tube was inserted postoperatively. The immediate postoperative course was uneventful, and the drains were removed on postoperative day 4. After full expansion of the right lung was observed on a chest roentgenogram, the patient was discharged.

After discharge, the patient started coughing and expectorating yellowish sputum. Physical examination of cardiac and abdomen revealed that they were normal, except for a scar on the abdominal skin. Infiltrative lesions were observed on a chest x-ray. No further investigation was performed for evaluating the biliary channels. Right inferior thoracotomy was performed at 17 days after laparotomy. Following the thoracotomy, phrenotomy was performed to investigate the dome of the liver. No bile leakage was detected, and a drain was inserted using a double-layer technique prior to closing the diaphragm. Since the lower lobe was partially damaged due to the penetrating injury, lower lobectomy was performed. The chest drains were removed on postoperative day 2, and the abdominal drain on day 5.

### Case 3

A 38-year-old man diagnosed with BBF at the centre where he had undergone a redo thoraco-phreno-laparotomy for hydatid disease of the liver and lung 4 months ago was admitted to our unit. No resectional surgery was performed in the previous operations. He presented with mild jaundice with total and conjuged bilirubin levels of 4.2 mg/dl and 0.71 mg/dl, respectively, and expectoration of yellow sputum for 3 weeks. A chest X-ray revealed that the volume of the right hemithorax was diminished with homogeneous density at the basal segments. MR cholangiography was not available at the time; therefore, percutaneous cholangiography was performed to evaluate the biliary channels. Right thoracotomy was performed for a third time. The lower lobe was almost totally damaged, and dissection between the liver and lung was extremely difficult. Therefore, we decided to perform lobectomy, and the lobe was resected along with a part of the diaphragm. The dome of the liver was closed with capitonage, and the diaphragm was closed primarily after inserting a subcostal drain. The patient was discharged on day 5 after the drains were removed.

## Discussion

Bronchobiliary fistula (BBF) is defined as an abnormal communication between the biliary system and bronchial tree. In 1850, Peacock described the first case of BBF in a patient presenting with liver hydatidosis [2]. BBF has also been reported as a congenital malformation; however, in most cases, it occurs following liver pathology, particularly parasitic infections. Several mechanisms are suggested for the development of BBF, including an inflammatory reaction in the subdiaphragmatic space with subsequent ruptures into the bronchial system and liver pathology which erodes the diaphragm leading a communication between bronchial tree and biliary channels. Although hydatid disease is still the leading cause of BBF, various other reasons are responsible for 20% of the reported cases (Table 1). These cases are attributable to the extensive surgical interventions and invasive procedures for the liver pathologies in recent years. Apart from anecdotal series that comprise a large number of cases [3,4], papers dealing with BBF rarely report more than a few cases. BBF is a serious complication associated with a high mortality and morbidity rate (12.2%) [5] and requires a well-planned management strategy. Despite the increasing number of reported cases, a widely accepted strategy for the management of BBF remains to be defined.

**Table 1:** Etiologies of BBF.

**Reasons of BBF**
Hydatid cyst
Trauma
Postsurgical states
Lithiasis in biliary trees
Subdiaphragmatic abscess
Cholecystitis/pancreatitis
Liver/biliary tree tumor
Radiofrequency ablation

Bile behaves as a strong irritant when present outside the biliary channel and gastrointestinal system. Early diagnosis requires a high index of suspicion, which can be easily determined by sputum analysis, and is essential to protect the lung tissue. An irritating cough and expectoration of yellowish sputum, termed bilioptysis, are the typical presentation of BBF. Sputum analysis verifies the levels of direct and indirect bilirubin, which doubtlessly indicate a direct communication between the biliary system and bronchial tree. Mild to moderate jaundice generally occurs in some cases. Generally, the clinical conditions of the patients are not encouraging due to an underlying chronic illness or previous surgical intervention. Patients are persuaded that the fistula should be treated without delay following intense supportive therapy which includes proper antibiotics and high calorie intake. Radiology reveals patch densities dominantly in the right lower lobe; however, these patches may also be present throughout the right lung and even in the left side [2,4,6]. Thoracic and upper abdominal CTs appear to be the best tools for the initial evaluation of lung and liver pathologies to plan further investigations.

Doubtlessly, whole treatment modalities fail if the obstruction in the biliary channel persists. Therefore, the first step following diagnosis is ensuring that the bile drains into duodenum without any obstruction. Among the numerous techniques employed for investigating the biliary channels, we prefer MR cholangiography for patients who probably have no pathology of the biliary tract. Endoscopic retrograde cholangiopancreatography (ERCP) may be an option for patients who present with a histology that suggests the need for endoscopic intervention. Fiberoptic bronchoscopy, which would reveal only yellowish bronchial secretion, plays no role in the initial assessment unless the patient's history implies intrabronchial pathology.

In those patients who present with pathology that interferes with the drainage of bile, the first intend should be the deliverance of the passage. This significantly reduces the amount of bile draining into the bronchial system [7,8]. A management plan depends on the primary illness and the findings on initial assessment. In patients with a short life expectancy in whom BBF developed due to a malignant disease, conservative approaches as a biliary stent or biliary decompression with endoscopic sphincterotomy provide sufficient palliation [9,10]. In patients in whom the obstruction is due to a benign disease, such as lithiasis or hydatid cyst, and sufficient drainage cannot be achieved, surgical intervention, which has a higher success rate, is recommended [2]. In such cases, peroperative cholangiography is recommended to confirm that the lumen is intact.

Persistent fistula with a patent biliary channel is an indication for thoracotomy. A delay results in further damage of the lung and requires lung resection. On thoracotomy, the fistula tract has to be entirely exposed, which necessitates exploration of the subdiaphragmatic area and the dome of the liver. The tract has to be completely resected, and the liver pathology is repaired to ensure no bile leakage. Tocchi reported good results with thoracoabdominal approach in 18 of 31 patients [11]. In our experience, we perform an inferior thoracotomy and expose the liver through the diaphragm without any difficulty. Therefore, we assume that thoracoabdominal approach should be reserved for the cases who requires a wide exposure over the fistula tract. We also routinely drain the subdiaphragmatic area in transthoracic extrapleural manner. The diaphragm is always closed by imbrication of the edges of the repaired liver and lower lobe. Chua et al. buttressed the repaired diaphragm with an intercostal muscle and pericardial fat pad [12]. It is accepted that anatomic lung resection (segmentectomy, lobectomy) should be considered for all cases, but as in case 3, conservative management should be kept in mind. Therefore, we assume that surgeons must adequately evaluate the parenchyma and seek a clear indication before proceeding with lung resection.

In conclusion, BBF is a condition that requires a high index of suspicion for diagnosis and a well-planned strategy for management. In the absence of these, it may cause a number of troublesome complications and even result in death. Further, lung resection in patients requiring thoracotomy may not be indicated in some cases.

## Authors' contributions

All authors have read and approved the final manuscript.

HE: Conceived the present study, participated in the analysis and drafting of the manuscript.

SO: Participated in the design, analysis and drafting of the manuscript.

GO: Participated in the analysis and drafting of the manuscript.

MK: Participated in the analysis and drafting of the manuscript.

CAK: Conceived the present study, participated in the analysis and drafting of the manuscript.
